# Hippocampal Inhibitory Interneuron‐Specific DREADDs Treatment Alters mTORC1‐4E‐BP Signaling and Impairs Memory Formation

**DOI:** 10.1111/jnc.70048

**Published:** 2025-03-24

**Authors:** Ziying Huang, Niaz Mahmood, Jean‐Claude Lacaille, Shane Wiebe, Nahum Sonenberg

**Affiliations:** ^1^ Department of Biochemistry McGill University Montreal Quebec Canada; ^2^ Goodman Cancer Institute Montreal Quebec Canada; ^3^ Department of Neuroscience and CIRCA University of Montreal Montreal Quebec Canada; ^4^ Integrated Program in Neuroscience McGill University, Montreal Neurological Institute Montreal Quebec Canada

**Keywords:** 4E‐BP, clozapine‐N‐oxide, hippocampus, interneurons, memory, mTORC1

## Abstract

Control of protein synthesis via the mechanistic target of rapamycin complex 1 (mTORC1) is essential for learning and memory. However, the cell‐type‐specific and spatiotemporal regulation of this pathway during memory formation is not well understood. In this study, we expressed artificial human muscarinic M3 [hM3D(Gq)] or M4 [hM4D(Gi)] designer receptors exclusively activated by designer drugs (DREADDs) in hippocampal CA1 excitatory or inhibitory neurons of adult mice. We studied the impact of clozapine‐N‐oxide (CNO), a synthetic DREADDs agonist, on the mTORC1 pathway and long‐term memory. hM3D(Gq) and hM4D(Gi) activate or inactivate, respectively, mTORC1 signaling in hippocampal interneurons, as indicated by the phosphorylation of its targets, eukaryotic initiation factor 4E‐binding proteins (4E‐BP1/2) and ribosomal protein S6 (S6). Activation of either hM3D(Gq) or hM4D(Gi) in mice immediately after training in memory tasks impaired long‐term memory formation in inhibitory, but not in excitatory neurons. The findings underscore the importance of activity‐dependent mTORC1–4E‐BP1/2 signaling in hippocampal inhibitory interneurons for memory formation.

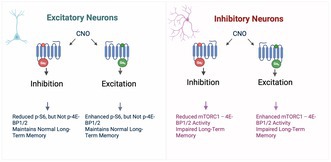

## Graphical abstract

We used advanced tools called DREADDs (Designer Receptors Exclusively Activated by Designer Drugs) to control the activity of inhibitory neurons versus excitatory neurons in the hippocampus, a region of the brain critical for learning and memory. By activating or inactivating these neurons after training, we found that disrupting their normal activity affected the brain's signaling pathway known as mTORC1, which is important for controlling protein synthesis in cells. This disruption impaired the ability to form long‐term object memories, specifically when inhibitory neurons were targeted, but not when excitatory neurons were targeted. Our results highlight the key role of hippocampal inhibitory neurons and their signaling in regulating memory, suggesting that precise timing of neuron activity is crucial for forming long‐term memories. These findings could have important implications for understanding brain disorders related to cognition.

**Abbreviations**
4E‐BPs: eukaryotic initiation factor 4E‐binding proteins
AAVs: Adeno‐associated viral vectors
AMPK: AMP‐activated protein kinase
AP: anterior–posterior
CA1: Cornus Ammonis‐1
Camk2α: calcium/calmodulin dependent protein kinase II alpha
cAMP: cyclic adenosine monophosphate
CCAC: Canadian council of animal care
CIHR: Canadian institutes of health research
ciPSI: cell‐type‐specific and drug‐inducible protein synthesis inhibition
cKO: conditional knockout
CNO: Clozapine‐N‐oxide
Cre: Cre recombinase
DI: discrimination index
DIO: double‐floxed inverted open reading frame
DKO: double knockout
DREADDs: Designer receptors exclusively activated by designer drugs
eIF4E: eukaryotic translation initiation factor 4E
ERK: ERK extracellular signal‐regulated kinase
FACC: Facility animal care committee (McGill University)
Gad65/Gad2: Glutamic acid decarboxylase 65
GC/ml: genome copies per milliliter
GPCRs: G‐protein‐coupled receptors
hM3: human muscarinic 3
hM4: human muscarinic 4
IEGs: immediate early genes
IF: immunofluorescence
i.p.: intraperitoneal
KO: knockout
mAChRs: muscarinic acetylcholine receptors
mg/kg: milligrams per kilogram
mg/ml: milligrams per milliliter
ML: medial‐lateral
mTORC1: mechanistic target of rapamycin complex 1
NE: novelty exploration
NOL: novel object location
NOR: novel object recognition
OPL: object place learning
PBS: phosphate‐buffered saline
PFA: paraformaldehyde
PHS: Public health service
PI3K: phosphoinositide 3‐kinase
Raptor: rapamycin (mTOR)‐binding protein
RRID: research resource identifier (see scicrunch.org)
RT: room temperature
S6K: ribosomal S6 kinase
scRNA‐Seq: single‐cell RNA‐seq
SYN1: synapsin 1
WT: wildtype

## 1 Introduction

Experience leads to memory formation via neuronal plasticity. The process of long‐term memory formation is dependent on *de novo* protein synthesis (Agranoff and Klinger 1964; Flexner et al. 1963; Shrestha, Ayata, et al. 2020; Shrestha, Shan, et al. 2020; Shrestha and Klann 2022). A key regulatory pathway for controlling *de novo* protein synthesis is the mechanistic target of rapamycin complex 1 (mTORC1) (Graber et al. 2013; Pereyra et al. 2018). By phosphorylating downstream targets, including ribosomal S6 kinase (S6K) and eukaryotic initiation factor 4E‐binding proteins (4E‐BP1/2), mTORC1 activates mRNA translation in the brain (Bekinschtein et al. 2007; Hara et al. 2002; Hay and Sonenberg 2004; Myskiw et al. 2008; Pereyra et al. 2018; Qi et al. 2010; Tang et al. 2002). Phosphorylation of S6K promotes translation and ribosome biogenesis, while phosphorylation of a translational repressor 4E‐BP1/2 releases it from the eukaryotic translation initiation factor 4E (eIF4E), allowing translation initiation (Beretta et al. 1996; Gingras et al. 2001; Hay and Sonenberg 2004; Prévôt et al. 2003). Substantial evidence exists that mTORC1 inhibition by rapamycin blocks downstream S6 and 4E‐BP1/2 phosphorylation, as well as long‐term memory formation (Beretta et al. 1996; Blundell et al. 2008; Lana et al. 2017; Muta et al. 2015; Parsons et al. 2006; Pearson et al. 1995).

Using brain cell‐type‐specific knockout (cKO) models, we previously demonstrated that conditional deletion of the genes encoding either 4E‐BP2 (to mimick mTORC1‐dependent 4E‐BP2 hyperphosphorylation) or Raptor, a subunit of mTORC1 (to suppress mTORC1 activity) in inhibitory neurons impairs long‐term object, location, and recognition memories (Huang et al. 2024). However, disrupting the mTORC1–4E‐BP2 axis in excitatory neurons, by deleting the genes encoding Raptor or 4E‐BP2, did not affect memory (Huang et al. 2024). A major drawback of general and conditional genetic KO models for memory studies is the lack of temporal regulation of gene deletion and the potential impact on brain development (Banko et al. 2007; Shinmyo et al. 2016). These confounding variables complicate the understanding of the role of mTORC1–4E‐BP2 in distinct stages of memory. To overcome these limitations, we employed a chemogenetic strategy to manipulate neuronal activity in a cell‐type‐specific manner. Using this approach, we investigated how spatiotemporal modulation of hippocampal neuronal activity affects mTORC1–4E‐BP1/2 signaling and influences spatial and recognition memory processes.

Neuronal plasticity in response to experience requires transcription of immediate early genes (IEGs), such as c‐Fos, and correlates with mTORC1 activity (Alberini and Kandel 2014; Cinalli et al. 2020; Lembke et al. 2011; Costa‐Mattioli and Monteggia 2013; Lembke et al. 2011; Niere and Raab‐Graham 2017; Villanueva et al. 2009). Therefore, we aimed to impact mTORC1 signaling in a bidirectional manner in the CA1 region of the hippocampus to assess its effects on long‐term memory formation. We chose to modulate neuronal activity by the designer receptors exclusively activated by designer drugs (DREADDs), which allow spatiotemporal control of neuronal activity in vivo (Roth 2016; Wess et al. 2013). G‐protein‐coupled receptor (GPCR)‐based DREADDs are artificially engineered muscarinic acetylcholine receptors (mAChRs) that are selectively activated by the synthetic ligand clozapine‐N‐oxide (CNO) (Wess et al. 2013). They exhibit transient activity and are not affected by endogenous muscarinic receptor ligands (Armbruster et al. 2007; Guettier et al. 2009; Wess et al. 2013). Muscarinic receptors are coupled to several G‐proteins: M1, M3, and M5 receptors are coupled to Gα_s/q_, and their activation results in enhanced neuronal excitability, whereas M2 and M4 are coupled to Gα_i/o_, and their activation reduces neuronal activity (Caulfield 1993; Durkee et al. 2019; Ham et al. 2024; Migeon et al. 1995). We employed two DREADDs featuring Cre‐dependent double‐floxed inverted open reading frames (DIO): AAV‐hSYN‐DIO‐hM3D(Gq)‐mCherry and AAV‐hSYN‐DIO‐hM4D(Gi)‐mCherry. These AAVs were injected into the hippocampal CA1 area and, since their expression requires Cre‐mediated recombination, their expression is restricted to hippocampal neurons expressing Cre recombinase (Roth 2016). Upon CNO treatment, activated hM3D(Gq) increases neuronal firing rates and activates neurons, while hM4D(Gi) suppresses neuronal firing and inhibits neuronal activity (Pati et al. 2019; Roth 2016; Stachniak et al. 2014; Wess et al. 2013). Receptor‐mediated changes in neuronal activity impact several signaling pathways—including mTORC1 and extracellular signal‐regulated kinase (ERK)—which control *de novo* protein synthesis, synaptic plasticity, and memory (Cabezudo et al. 2021; Rogan and Roth 2011).

DREADDs enable temporal manipulation of mTORC1 signaling in specific cell types (Cabezudo et al. 2021; Rogan and Roth 2011). For example, hM3D(Gq) activation causes mTORC1 signaling and protein synthesis stimulation both in cell lines and in vivo (Cabezudo et al. 2021; Gong et al. 2023; Shrestha, Ayata, et al. 2020; Shrestha, Shan, et al. 2020; Wu et al. 2020). Because hM4D(Gi) signaling suppresses neuronal activity, it is expected to result in decreased mTORC1 signaling, indicated by diminished phosphorylation of S6 and 4E‐BP1/2 (Beretta et al. 1996; Pearson et al. 1995). We targeted the hippocampus—which is critical for memory encoding and retrieval—using DREADDs to manipulate neuronal activity in *Gad2‐Cre* (inhibitory neurons) and *Camk2a‐Cre* (excitatory neurons) mice. We found that post‐training activation [by hM3D(Gq)] or inhibition [by hM4D(Gi)] of hippocampal inhibitory neurons, but not excitatory neurons, bidirectionally regulated mTORC1 signaling and disrupted memory formation in mice. These effects were observed across object place learning (OPL), novel object location (NOL) and novel object recognition (NOR) tasks. These tasks rely heavily on forebrain and specifically hippocampal function. Taken together, our findings indicate that stimulation of inhibitory interneurons elicits mTORC1–4E‐BP2 signaling, which is crucial for memory formation.

## 2 Materials and Methods

### 2.1 Reagent and Resource

**Table:** 

Antibodies and chemicals	Source	Catalog Numbers	RRIDs
p‐4E‐BP1/2(T37/46) Rabbit antibody	Cell Signaling Technology	2855	AB_560835
p‐S6(S240/244) Rabbit antibody	Cell Signaling Technology	5364	AB_10694233
mCherry Mouse/Rabbit antibody	Abcam	125096/167453	AB_11133266/AB_2571870
CaMK2α(6G9) Mouse antibody	Cell Signaling Technology	50 049	AB_2721906
GAD67 Mouse antibody	Millipore	MAB5406	AB_2278725
AAV‐hSYN‐DIO‐hM3D(Gq)‐mCherry	Addgene	44361‐AAV9	Addgene_44361
AAV‐hSYN‐DIO‐hM4D1(Gi)‐mCherry	Addgene	44362‐AAV1	Addgene_44362
Alexa Fluor 488 Goat Anti‐Rabbit Secondary Antibody	Thermo Fisher Scientific	11034	AB_2576217
Alexa Fluor 546 Donkey Anti‐Mouse Secondary Antibody	Thermo Fisher Scientific	10036	AB_11180613
Hoechst 33342 Trihydrochloride, Trihydrate	Life Technologies	3570	AB_3675235
Paraformaldehyde	Sigma‐Aldrich	P6148	NA
CNO	TOCRIS	6329	NA
Normal Goat serum	Abcam	ab7481	NA
Glass Antifade Mountant	Thermo Fisher Scientific	s36972	NA

Mouse models	Source	Catalog Numbers	RRIDs
*Camk2a‐Cre*	Jackson Laboratory	005359	IMSR_JAX:005359
*Gad65‐Cre*	Jackson Laboratory	010802	IMSR_JAX:010802
C57BL/6J mice	Jackson Laboratory	000664	IMSR_JAX:000664
*eif4ebp1*KO	Jackson Laboratory (Tsukiyama‐Kohara et al. 2001)	**031225**	**IMSR_JAX:031225**
*eif4ebp2* KO	Jackson Laboratory (Banko et al. 2005)	**031507**	**IMSR_JAX:031507**
*eif4ebp1/eif4ebp2* DKO	Generated in the Sonenberg lab (Le Bacquer et al. 2007)	NA	NA

Devices and software	Source	Catalog numbers	RRIDs
Stereotaxic instrument	Intellibio Innovation	68 043	NA
Elite Syringe Pumps	Harvard Apparatus	HA1100D	NA
33G Needles	Hamilton	14‐785‐715	NA
Hamilton Gastight and Microliter Syringe	Hamilton	14‐813‐310	NA
LSM 880 with Airyscan Confocal Laser Scanning Microscope	Carl Zeiss	NA	SCR_020925
Zen Microscopy software	Carl Zeiss	NA	SCR_013672
Zen Blue Edition	Carl Zeiss	NA	**SCR_013672**
Prism 10	GraphPad Software	NA	SCR_002798

### 2.2 Animal Models

Mice expressing *Camk2a‐Cre* (#005359) and *Gad2‐Cre* (#010802) were purchased from the Jackson Laboratory (JAX) on a C57BL/6J background or backcrossed to C57BL/6J mice (#000446) for more than 10 generations. Mice were group‐housed (2–5 per cage) in the Goodman Cancer Institute animal facility and maintained on a standard 12/12 h light/dark cycle (7:00–19:00 light period). Mice were always given *ad libitum* access to food and water. The housing room and testing room maintained a consistent temperature (around 22°C) and low noise levels. At weaning (postnatal day 21), mice were ear‐notched for identification. To verify Cre‐recombinase expression, DNA was extracted from ear‐punch samples and subjected to PCR amplification using the following primers: *Cre*‐F (5′‐GATTGCTTATAACACCCTGTTACG‐3′) and *Cre*‐R (5′‐GTAAATCAATCGATGAGTTGCTTCA‐3′). Mice (male) were 2 months old at the time of surgery and around 3 months old at the time of memory tests. Experimental mice were handled 2 days before the behavioral experiments. All experiments involving animals were approved by the McGill University Facility Animal Care Committee (FACC) regulations, which follow the guidelines established by the Canadian Council on Animal Care (CCAC) and NIH Office of Laboratory Animal Welfare (OLAW). Public health service (PHS) assurance number for McGill University is F‐16‐00005(A5006‐01).

### 2.3 Designer Receptors Exclusively Activated by Designer Drugs (DREADDS) Injection

Neuronal‐specific expression of the DREADDs were obtained using adeno‐associated viral vectors (AAVs) encoding either the hM3D(Gq) or hM4D(Gi) containing a Double‐Floxed Inverted Open Reading Frame (DIO) and were fused to an mCherry tag for fluorescence detection. The hM4D(Gi) and hM3D(Gq) constructs under the control of the human *Synapsin1* (SYN1) promoter were described (Krashes et al. 2011). AAV‐hSYN‐DIO‐hM3D(Gq)‐mCherry (Addgene 44 361‐AAV9, 1.8 × 10^13^ GC/mL (Genome copies per milliliter)) or AAV‐hSYN‐DIO‐hM4D1(Gi)‐mCherry (Addgene 44 362‐AAV1, 2.3 × 10^13^ GC/ml) were diluted 1:50 with sterile saline. Mice were injected with 1 μL of AAV vector into each hippocampus. Following a 3‐week recovery period, mice were subjected to behavioral experiments. To activate hM4D(Gi)‐ or hM3D(Gq)‐DREADDs for decreasing and enhancing neuron activity, the synthetic drug clozapine N‐oxide (CNO) was given by i.p. injection at 0.04 mg/mL (0.1 mg/kg), immediately after each training session of behavioral experiments. A total of 92 mice were injected with AAVs, 19 of which were assigned to the CaMK2A‐hM4D(Gi) group, among which 10 received saline and 9 received CNO. In the GAD2‐hM4D(Gi) experiment, 30 mice were injected with AAV, among which 15 received saline and 15 received CNO. For the GAD2‐hM3D(Gq) group, 19 mice were initially included, with 9 mice in the saline group and 10 mice in the CNO group (one mouse with a total exploration time of 1.15 s during the NOL test was excluded from all tests). In the CaMK2A‐hM3D(Gq) experiment, 24 mice were used, with 12 mice in the saline and 12 in the CNO groups.

### 2.4 Stereotactic Surgeries

Mice were anesthetized with isoflurane‐infused oxygen (1.5%–2%) and received 5 mg/kg carprofen and 0.9% saline before surgery to prevent dehydration during the procedure and to provide pain relief as soon as the mice recovered from the anesthetic. Mice were placed on a heated pad where the head was secured on a mouthpiece between ear pins. A small patch of fur was shaved from the head, and the skin was disinfected with Isobetadine. After confirming loss of pain reflexes (by pinching posterior paws), a small incision (3 mm) was made in the scalp using a scalpel blade. The surface of the skull was swabbed with hydrogen peroxide to locate bregma and disinfect the surgery area. The stereotactic apparatus was zeroed at bregma, and the coordinates anterior–posterior (AP): −1.9 mm and medial‐lateral (ML): ±1 mm were used to identify the CA1 hippocampal region. Using a small drill, holes were made in the skull where a 33G needle was lowered to a depth of 1.4 mm to deliver the AAV. Following surgery, mice were monitored for respiratory and reflexes until they fully recovered from anesthesia. During the first 3 days post‐surgery, their general appearance, activity levels, and the surgical site were closely observed for any signs of complications. Veterinary care staff in the animal facility continued to monitor and evaluate the mice throughout our experimental period.

### 2.5 Immunofluorescence

A separate group of mice (*n* = 4 per experimental group, 32 in total) that received AAV injections but did not undergo behavioral experiments were anesthetized with 2% isoflurane in oxygen. The mice were then perfused with ice‐cold 1× phosphate‐buffered saline (PBS), followed by 4% paraformaldehyde (PFA, Sigma‐Aldrich, P6148). Brains were collected and fixed in PFA at 4°C. Three days prior to the immunofluorescence staining procedure, brain samples were transferred to a 30% sucrose solution in 1× PBS for cryoprotection. Mouse hippocampal coronal sections (20 μm) were collected using a cryostat. Sections were placed in 10 mM boiling sodium citrate buffer for 20 min and washed with 1× PBS. Sections were blocked in blocking buffer (10% normal goat serum and 0.1% Triton‐100 in PBS) for 1.5 h at room temperature, followed by primary antibody incubation (diluted in 5% normal goat serum) at 4°C overnight. Antibodies used in this study are as follows: mCherry, Abcam #125096, 1:1000; CaMK2A, Cell Signaling Technology #50049, 1:400; GAD67, Millipore MAB5406, 1:5000; p‐S6 Ribosomal Protein (Ser240/244), Cell Signaling Technology, #5364, 1:200; p‐4E‐BP1/2 (Thr37/46), Cell Signaling Technology, #2855, 1:1000. Sections were washed with 1× PBS 3 times, then incubated in Alexa Fluor 488 goat anti‐rabbit IgG (Thermo Fisher Scientific, #11034, 1:400), Alexa Fluor 546 donkey anti‐mouse IgG (Thermo Fisher Scientific, #10036, 1:400) and Hoechst 33342, trihydrochloride, trihydrate (Life Technologies, #3570, 1:5000) in blocking buffer for 1 h at RT. Brain slices were mounted with Glass Antifade Mountant (Thermo Fisher Scientific, #s36972) and imaged with a ZEISS LSM880 laser scanning confocal microscope. Images were analyzed using Zen Lite software (ZEISS Microscopy) under consistent exposure and gain settings across groups. The integrated density of the immunofluorescence signal was measured from 10 hippocampal cells (either excitatory or inhibitory neurons) per animal (*n* = 4 per treatment group). Mean values for each animal were calculated, and the relative integrated density was determined by normalizing to the saline control group using Zeiss Zen software.

### 2.6 Behavioral Testing

Mice were handled for 3 consecutive days before habituation, allowing them to explore the experimenter's hand for 2 min once per day. Behavioral experiments were conducted between 7 a.m. and 3 p.m. Prior to each session, mice were habituated to the room under dim lighting for at least 30 min. A two‐day rest period was provided between the novel object location and novel object recognition tests. Mice were introduced to test boxes in a counterbalanced manner, and the order of testing was randomized to prevent bias. The experimenter was blinded to the mice's treatments during data analysis and scoring (manual). Manual scoring included sniffing and climbing on the objects as exploration time, while sitting or standing on the objects was excluded. The discrimination index (DI) was calculated as the difference between the novel and familiar object exploration times, divided by the total exploration time, then multiplied by 100. The index of novelty exploration (NE) was calculated as the total exploration time on Day 2/Day 1 × 100.

### 2.7 Novel Object Location (NOL)

Mice were habituated twice to an empty white box (50 cm × 50 cm × 30 cm), once in the morning (between 7 a.m.–11 a.m.) and once in the afternoon (12 noon–3 p.m.) for 10 min each session. The following day, mice underwent two training sessions in the same boxes with two identical objects. Training was performed once in the morning and again in the afternoon for 10 min each and was repeated on a second day for a total of four sessions. Vehicle or CNO was i.p. injected after each training session. The day after training, memory retention was tested by moving one object to a new location. After 24 h, mice were reintroduced into the same box and allowed to explore the objects freely for 10 min. Testing sessions were recorded with an overhead camera.

### 2.8 Novel Object Recognition (NOR)

Using the same setup as NOL, mice were habituated in the morning (between 7 a.m.–11 a.m.) for 10 min. The following day (training Day 1), they were trained once in the morning for 10 min to explore two identical objects. The training was repeated the next day (training Day 2) for a total of two sessions. Vehicle or CNO was i.p. injected after each training session. On the final day, one object was replaced by a novel, unfamiliar object with a different shape. After 24 h, mice were allowed to explore the objects for 10 min. Both training and testing sessions were recorded with an overhead camera.

### 2.9 Object Place Learning (OPL)

OPL was assessed using data from the two sessions of NOR on training Day 1 and training Day 2. If the mice retained memory of the objects, their total exploration time would be reduced on Day 2 compared to Day 1.

### 2.10 Statistical Analysis

All graphing and statistical analysis were performed using GraphPad Prism 10. No statistical methods were used to pre‐determine the sample size. The sample size (> 9 mice per group) was chosen based on prior studies that employed long‐term memory tests and the sample sizes commonly used in the C57/BL/6 J background strain (Banko et al. 2005; Huang et al. 2024; Shrestha, Shan, et al. 2020; Zhu et al. 2018). After scoring and data analysis, one mouse from the GAD2‐hM3D(Gq) group was excluded due to a low total exploration time of 1.15 s, below the recommended cut‐off of 3 s for exclusion (Vogel‐Ciernia and Wood 2014). In the CaMK2A‐hM3D(Gq) experiment, two mice were excluded as outliers, as determined using the GraphPad Grubbs' test (α = 0.05). Normality was assessed using the Shapiro–Wilk test, due to its high sensitivity in small sample sizes (9–15 animals per group in our behavioral study). In GAD2‐hM3D(Gq) mice, the saline and CNO groups from the OPL and NOR tests did not pass the normality and lognormality test (Shapiro–Wilk test, *α* = 0.05). Therefore, a non‐parametric Mann–Whitney U test was used for statistical analysis. Additionally, when the standard deviations between groups were significantly different (F‐test, *p* < 0.05), a Welch's corrected *t*‐test was performed. Bar graph data are presented as mean ± standard error of the mean (SEM). DI are displayed as box and whisker plots, with the box representing the interquartile range, the line in the middle indicating the median, and the “+” indicating the mean. The whiskers represent the minimum and maximum data points. The “#” symbol represents the *p*‐value from a one‐sample *t*‐test comparing the dataset mean to a hypothetical value (indicated by a grid line). Data were analyzed using ANOVA, followed by post hoc comparisons when appropriate. The asterisk “*” represents *p*‐values from an unpaired student's *t*‐test. Statistical significance was set at *p* < 0.05 (ns: *p* > 0.05, *p ≤ 0.05, **p ≤ 0.01, ***p ≤ 0.001, ****p ≤ 0.0001).

## 3 Results

### 3.1 Activation of hM4D(Gi) and hM3D(Gq) DREADDs in Hippocampal Interneurons Bidirectionally Modulates mTORC1 Activity and Phosphorylation of 4E‐BP1/2

**FIGURE 1:**
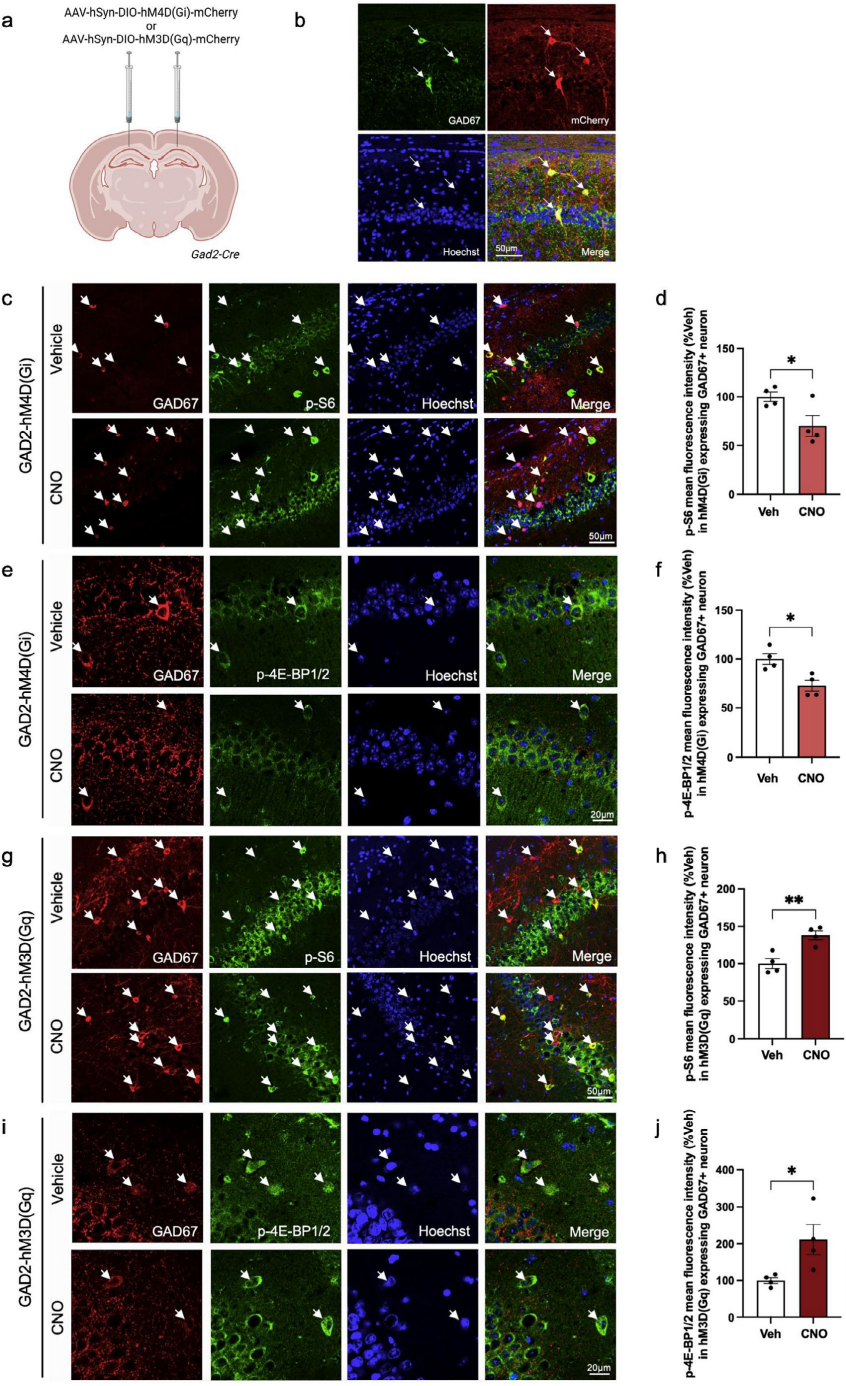
Hippocampal DREADDs expression and bidirectional manipulation of mTORC1 activity in *Gad2‐Cre* mice via hM4D(G_i_) and hM3D(G_q_). (a) Schematic of AAV injection into the CA1 hippocampus of *Gad2‐Cre* mice. (b) mCherry fluorescence in the CA1 hippocampus 3 weeks following injection. GAD67 (green) and mCherry (red) fluorescence overlap (yellow, merged image). Scale bar 50 μm. (c) Fluorescence analysis of p‐S6 (S240/244) (green) in the hippocampus Gad67^+^ neuron (red) in GAD2‐hM4D(Gi) + CNO (number of animals = 4) vs. GAD2‐hM4D(Gi) + saline (number of animals = 4). (d) Quantification (integrated density) of p‐S6 (240/244) from (c). (e) Fluorescence analysis of p‐4E‐BP1/2 (T37/46) (green) in the hippocampus Gad67^+^ neuron (red) in GAD2‐hM4D(Gi) + CNO (number of animals = 4) vs. GAD2‐hM4D(Gi) + saline (number of animals = 4). (f) Quantification (integrated density) of p‐4E‐BP1/2 (T37/46) from (e). (g) Fluorescence analysis of p‐S6 (240/244) (green) in the hippocampus Gad67^+^ neuron (red) in GAD2‐hM3D(Gq) + CNO (number of animals = 4) vs. GAD2‐hM3D(Gq) + saline (number of animals = 4). (h) Quantification (integrated density) of p‐S6 (240/244) from (g). (i) Fluorescence analysis of p‐4E‐BP1/2 (T37/46) (green) in the hippocampus Gad67^+^ neuron (red) in GAD2‐hM3D(Gq) + CNO (number of animals = 4) vs. GAD2‐hM3D(Gq) + saline (number of animals = 4). (j) Quantification (integrated density) of p‐4E‐BP1/2 (T37/46) from (i). Cell nuclei are stained with Hoechst (blue). White arrows indicate GABAergic (i.e., GAD67) inhibitory neurons. Scale bar represents 50 μm (c and g) and 20 μm (e and i). *p < 0.05, **p < 0.01, calculated with an unpaired *t*‐test. Data are presented as mean ± SEM.

We first examined whether mTORC1 signaling is temporally controlled by hM4D(Gi) and hM3D(Gq) DREADDs in hippocampal inhibitory neurons. mCherry‐tagged hM4D(Gi) or hM3D(Gq) DREADDs containing a DIO were packaged into AAV and injected into the hippocampus of 2‐month‐old *Gad2‐Cre* mice (Figure 1a). After 3 weeks, mCherry expression was confirmed in GAD67^+^ inhibitory neurons (Figure 1b). To activate DREADDs, CNO was administered via intraperitoneal (i.p.) injection at 0.1 mg/kg (Alexander et al. 2009; Rogan and Roth 2011; Vaidyanathan et al. 2021). Mice were anesthetized 1 h after CNO administration, and then transcardially perfused with 4% paraformaldehyde before collecting the brains. Immunofluorescence (IF) was performed to measure phosphorylation of the two main downstream mTORC1 targets: p‐S6 (Ser240/244) and p‐4E‐BP1/2 (Thr37/46). We confirmed the specificity of the p‐4E‐BP1/2 antibody by IF on wildtype (WT), 4E‐BP1 knockout (KO), 4E‐BP2 KO, and 4E‐BP1/2 double knockout (DKO) mice. No signal was observed in the hippocampus of DKO mice compared to WT (Figure S1). 4E‐BP2 is the major 4E‐BP isoform in the brain, primarily expressed in neurons, and is essential for long‐term memory (Aguilar‐Valles et al. 2021; Banko et al. 2007, 2005). We detected p‐4E‐BP2 in the brain of 4E‐BP1 KO mice, whereas p‐4E‐BP1 signal was weak in the 4E‐BP2 KO brain, as expected from the literature (Aguilar‐Valles et al. 2021; Banko et al. 2005) (Figure S1).

In *Gad2‐Cre* mice expressing hM4D(Gi) [GAD2‐hM4D(Gi)], CNO treatment led to a similar magnitude of reduction in p‐S6 (Ser240/244) (29.92% ± 11.64%, cell number = 40, mouse number = 4, *p* = 0.0424, *t* = 2.570, df = 6, unpaired student's *t*‐test) and p‐4E‐BP1/2 (Thr37/46) (27.25% ± 7.93%, cell number = 40, number of animals = 4, *p* = 0.0139, *t* = 3.437, df = 6, unpaired student's *t*‐test) levels in hippocampal inhibitory neurons compared to vehicle control (Figure 1c–f). Conversely, for *Gad2‐Cre* mice injected with hM3D(Gq) [GAD2‐hM3D(Gq)], CNO treatment resulted in an increase in p‐S6 (Ser240/244) (38.15% ± 9.053%, cell number = 33–40, number of animals = 4, *p* = 0.0056, *t* = 4.214, df = 6, unpaired student's *t*‐test) and p‐4E‐BP1/2 (Thr37/46) (111.6% ± 41.79%, cell number = 40, number of animals = 4, *p* = 0.0370, *t* = 2.670, df = 6, unpaired student's *t*‐test) in inhibitory neurons compared to vehicle control (Figure 1g–j). The results demonstrate that mTORC1 signaling is bidirectionally controlled in inhibitory neurons of the CA1 hippocampus using hM4D(Gi) and hM3D(Gq) DREADDs.

### 3.2 hM4D(Gi) and hM3D(Gq) DREADDs Modulation in Inhibitory Neurons After Learning Prevents Hippocampal‐Dependent Memory Formation

**FIGURE 2:**
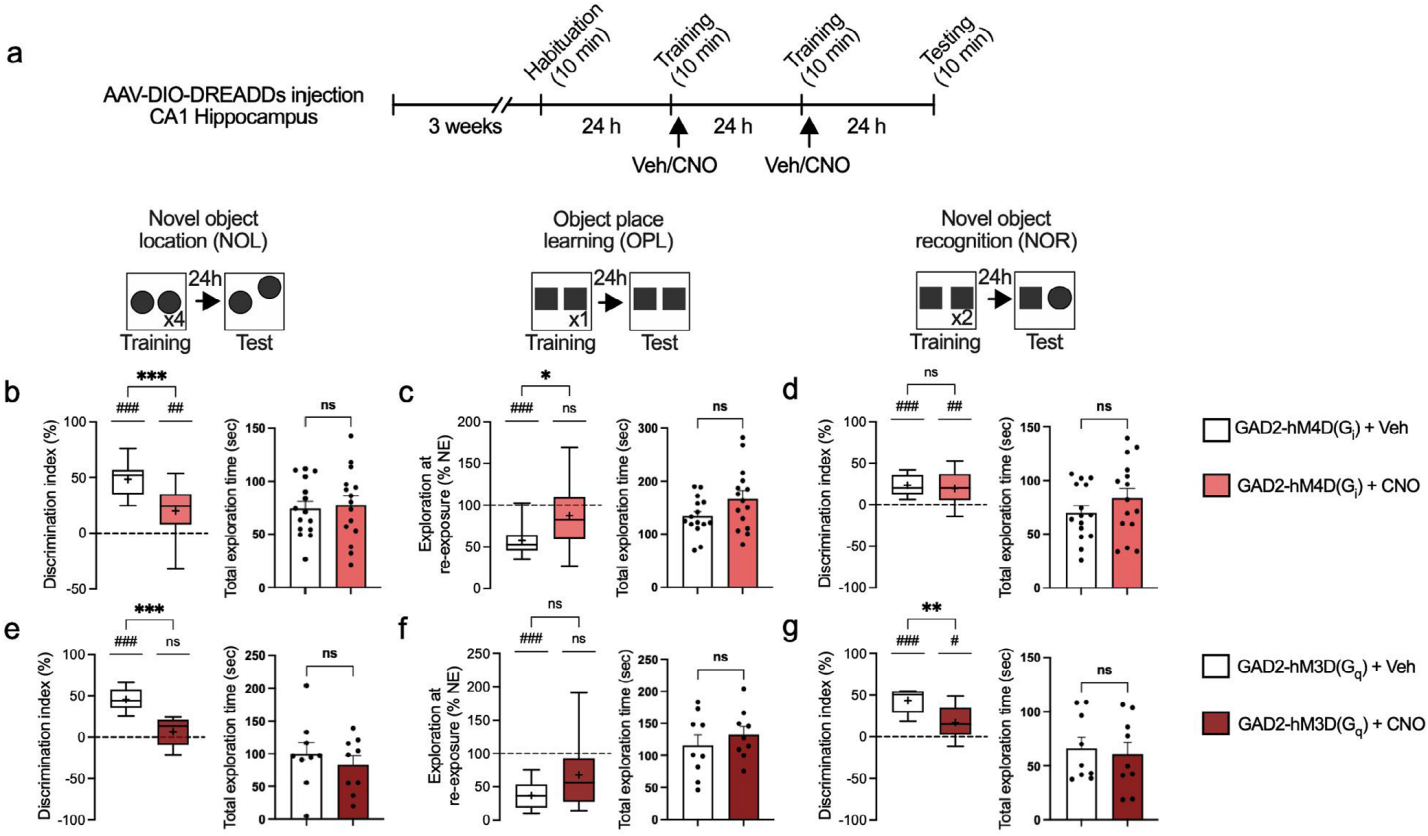
Post‐training activation of hM4D(G_i_) and hM3D(G_q_) DREADDs in inhibitory neurons disrupts hippocampus‐dependent memory formation. (a) Schematic of the timeline and experimental design with *Gad2‐Cre* mice. Mice underwent stereotactic surgery where the DREADDs were infused bilaterally into the CA1 hippocampus. Following 3 weeks of recovery and viral expression, mice were habituated to the testing apparatus. On subsequent days, mice were subjected to a series of training immediately followed by an i.p. injection of saline or CNO. Mice were tested for memory ability on the final day according to the protocol. (b–d) Memory measured by discrimination index of object exploration and total exploratory behavior during testing in GAD2‐hM4D(Gi) + CNO (NOL number of animals =15; OPL number of animals = 15; NOR number of animals = 15) vs. GAD2‐hM4D(Gi) + saline (NOL number of animals = 15; OPL number of animals = 15; NOR number of animals = 15). (e–g) Memory measured by discrimination index of object exploration and total exploratory behavior during testing in GAD2‐hM3D(Gq) + CNO (NOL number of animals = 9; OPL number of animals = 9; NOR number of animals = 9) vs. GAD2‐hM3D(Gq) + saline (NOL number of animals = 9; OPL number of animals = 9; NOR number of animals = 9). *p < 0.05, **p < 0.01, ***p < 0.001, ns not significant, calculated by an unpaired *t*‐test. ^#^ *p* < 0.05, ^##^ *p* < 0.01, ^###^ *p* < 0.001, ns not significant, calculated by a one sample *t*‐test. Data are presented with box and whisker plots where “+” indicates the mean. Dashed line indicates no discrimination of objects (i.e., memory impairment).

We recently demonstrated that inhibitory neuron‐specific deletion of either Raptor (inhibiting mTORC1) or 4E‐BP2 (mimicking mTORC1 overactivation and 4E‐BP2 hyperphosphorylation) engenders deficits in long‐term memory formation (Huang et al. 2024). It is noteworthy, however, that in utero and early developmental gene deletion may cause compensatory changes and introduce confounding factors, such as alterations in cell number, dendritic complexity, and neurite length (Gong et al. 2015; Lin et al. 2016; Sharma et al. 2021; Shinmyo et al. 2016; Wiebe et al. 2023; Yu et al. 2023). To overcome these limitations, we activated hM4D(Gi) or hM3D(Gq)‐coupled DREADDs in the hippocampus of *Gad2‐Cre* mice immediately after the training session in memory tasks to alter mTORC1 signaling and test the effects on memory formation (Figure 2a).

Modulating the activity of inhibitory neurons, either through suppression via hM4D(Gi) or enhancement via hM3D(Gq), altered mTORC1–4E‐BP1/2 signaling and engendered memory deficits in the novel object location (NOL) task, as indicated by changes in the discrimination index (DI) between CNO treatment and the control group [GAD2‐hM4D(Gi): *p* = 0.0007, number of animals = 15 for each group, *t* = 3.803, df = 28; GAD2‐hM3D(Gq): *p* = 0.0034, number of animals = 9 for each group, *t* = 3.430, df = 16, unpaired student's *t*‐test](Figure 2b,e). In the object place learning (OPL) task, control mice with intact learning and memory abilities typically show a decrease in exploration at reexposure (NE), as they spend less time exploring familiar objects upon reexposure. When inhibitory neurons were manipulated using either hM4D(Gi) or hM3D(Gq), OPL was impaired (Figure 2c,f). The impairments are supported by the absence of a decrease in NE relative to baseline in CNO‐treated groups [GAD2‐hM4D(Gi) + Saline, *p* < 0.0001, *t* = 9.263, df = 14; GAD2‐hM4D(Gi) + CNO, *p* = 0.1634, *t* = 1.471, df = 14; GAD2‐hM3D(Gq) + Saline *p* = 0.0013, *t* = 4.857, df = 8; GAD2‐hM3D(Gq) + CNO *p* = 0.0712, *t* = 2.079, df = 8, unpaired student's *t*‐test]. Suppression of inhibitory neurons via hM4D(Gi) significantly disrupted OPL, as indicated by a significant change in NE [GAD2‐hM4D(Gi): *p* = 0.0392, *t* = 2.216, df = 18.75, unpaired student's *t*‐test](Figure 2c). Although activation of inhibitory neurons via hM3D(Gq) using CNO did not produce a statistically significant difference in NE compared to the saline group [GAD2‐hM3D(Gq): *p* = 0.5457, Mann–Whitney U test](Figure 2f), object place learning and memory was still considered impaired due to the lack of NE reduction as explained earlier. In the novel object recognition (NOR) test, the activation [GAD2‐hM3D(Gq), *p* = 0.0244, Mann–Whitney U test], but not inhibition [GAD2‐hM4D(Gi), *p* = 0.9497, *t* = 0.06371, df = 28, unpaired student's *t*‐test], of CA1 inhibitory neuronal activity impaired memory (Figure 2d,g). This difference between the results of the NOR test and other tests like NOL and OPL may be due to the involvement of other regions, such as the dentate gyrus and cortex, in recognition memory (Barker and Warburton 2011; Dees and Kesner 2013). Barker and Warburton (2011) described the combined contributions of the hippocampus and its surrounding cortical regions in recognition memory tasks, suggesting that compensatory mechanisms might mitigate hippocampal dysfunction and NOR memory impairments. Additionally, the chemogenetic manipulation did not impair total exploratory behavior, confirming that the DREADDs interventions selectively target memory without affecting the baseline activity of the mouse (Figure 2b–g, right panels).

Our data demonstrate that hM4D(Gi) and hM3D(Gq)‐coupled DREADDs disrupt long‐term memory when activated in hippocampal inhibitory interneurons immediately after training, likely but not exclusively (see below) due to modulation of mTORC1–4E‐BP1/2 signaling during memory formation (Huang et al. 2024).

### 3.3 Activation of hM4D(Gi) and hM3D(Gq) DREADDs in Hippocampal Excitatory Neurons Alters mTORC1 Activity but Not 4E‐BP1/2 Phosphorylation

**FIGURE 3:**
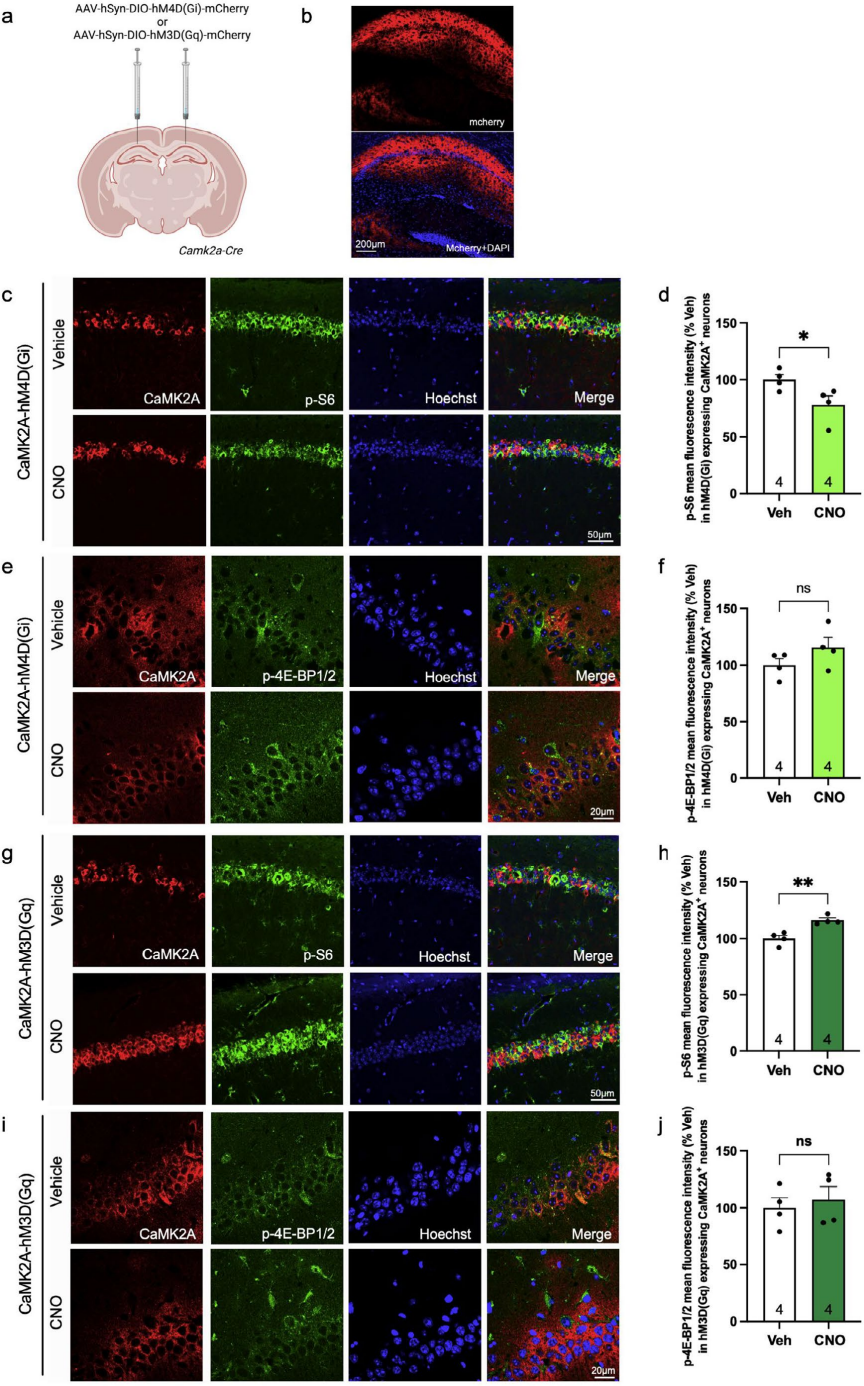
Hippocampal DREADDs expression and bidirectional manipulation of mTORC1 activity in *Camk2a‐Cre* mice by hM4D(G_i_) and hM3D(G_q_). (a) Schematic of AAV injection into the CA1 hippocampus of *Camk2a‐Cre* mice. (b) mCherry fluorescence in the CA1 hippocampus 3 weeks following injection. Scale bar 200 μm. (c) Fluorescence analysis of p‐S6 (240/244) (green) in the hippocampus CaMK2A^+^ neurons (red) in CaMK2A‐hM4D(Gi) + CNO (number of animals = 4) vs. CaMK2A‐hM4D(Gi) + saline (number of animals = 4). (d) Quantification (integrated density) of p‐S6 (240/244) from (c). (e) Fluorescence analysis of p‐4E‐BP1/2 (T37/46) (green) in the hippocampus CaMK2A^+^ neurons (red) in CaMK2A‐hM4D(Gi) + CNO (number of animals = 4) vs. CaMK2A‐hM4D(Gi) + saline (number of animals = 4). (f) Quantification (integrated density) of p‐4E‐BP1/2 (T37/46) from (e). (g) Fluorescence analysis of p‐S6 (240/244) (green) in the hippocampus CaMK2A^+^ neurons (red) in CaMK2A‐hM3D(Gq) + CNO (number of animals = 5) vs. CaMK2A‐hM3D(Gq) + saline (number of animals = 5). (h) Quantification (integrated density) of p‐S6 (240/244) from (g). (i) Fluorescence analysis of p‐4E‐BP1/2 (T37/46) (green) in the hippocampus CaMK2A^+^ neurons (red) in CaMK2A‐hM3D(Gq) + CNO (number of animals = 4) vs. CaMK2A‐hM3D(Gq) + saline (number of animals = 4). (j) Quantification (integrated density) of p‐4E‐BP1/2 (T37/46) from (i). Cell nuclei are stained with Hoechst (blue). The pyramidal cell layer indicates CA1 excitatory (i.e., CaMK2A) neurons. Scale bar represents 50 μm (c and g) and 20 μm (e and i). *p < 0.05, **p < 0.01, calculated with an unpaired *t*‐test. Data are presented as mean ± SEM.

To investigate the impact of DREADDs activation on neuronal mTORC1 activity in excitatory neurons, we injected hM4D(Gi) or hM3D(Gq)‐coupled DREADDs into the hippocampus of *Camk2a‐Cre* mice. After 3 weeks, mCherry signal was observed in the CA1 region of the hippocampus (Figure 3b), demonstrating successful delivery of the DREADDs. CNO was administered via i.p. injection, and 1 h later, brain samples were collected for immunofluorescence. In *Camk2a‐Cre* mice injected with hM4D(Gi) [CaMK2A‐hM4D(Gi)], CNO treatment resulted in a small reduction of p‐S6 (Ser240/244) (21.83% ± 8.909%, cell number = 40, number of animals = 4, *p* = 0.0498, *t* = 2.450, df = 6, unpaired student's *t*‐test) in CA1 CaMK2A^+^ neurons (Figure 3c,d). No significant difference in the levels of p‐4E‐BP1/2 (Thr37/46) (15.39% ± 10.51%, cell number = 40, number of animals = 4, *p* = 0.1935, *t* = 1.464, df = 6, unpaired student's *t*‐test) was observed in hippocampal excitatory neurons in CNO versus vehicle‐treated groups (Figure 3e,f). Similarly, in *Camk2a*‐*Cre* mice injected with hM3D(Gq) [CaMK2A‐hM3D(Gq)], CNO induced a small increase in p‐S6 (Ser240/244) (16.00% ± 3.435%, cell number = 38–40, number of animals = 4, *p* = 0.0035, *t* = 4.658, df = 6, unpaired student's *t*‐test) in CA1 CaMK2A^+^ neurons (Figure 3g,h), but no change in p‐4E‐BP1/2 (Thr37/46) (7.316% ± 14.26%, cell number = 40, number of animals = 4, *p* = 0.6263, *t* = 0.5129, df = 6, unpaired student's *t*‐test) level was detected compared to vehicle control (Figure 3i,j). The data demonstrate that hM4D(Gi) and hM3D(Gq)‐coupled DREADDs affect 4E‐BP1/2 phosphorylation in inhibitory but not excitatory neurons.

### 3.4 hM3D(Gq) and hM4D(Gi) DREADDs in Excitatory Neurons Do Not Affect Hippocampal‐Dependent Memory Formation After Learning

**FIGURE 4:**
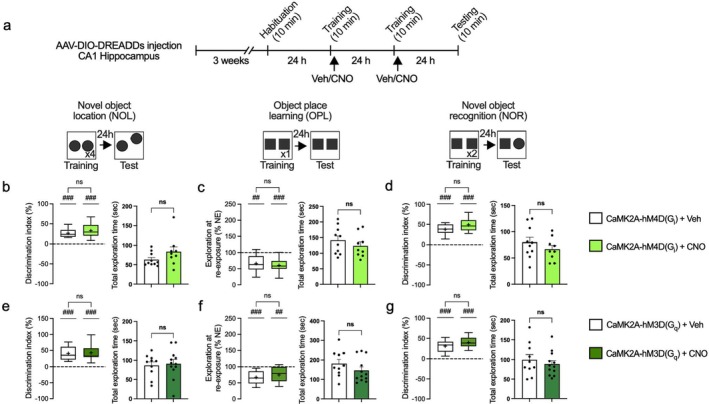
Post‐training activation of hM4D(G_i_) and hM3D(G_q_) DREADDs in excitatory neurons does not affect hippocampus‐dependent memory formation. (a) Schematic of the timeline and experimental design with *Camk2‐Cre* mice. (b–d) Memory measured by discrimination index of object exploration, and total exploratory behavior during testing in CaMK2A‐hM4D(Gi) + CNO (NOL number of animals = 9; OPL number of animals = 9; NOR number of animals = 9) vs. CaMK2A‐hM4D(Gi) + saline (NOL number of animals = 10; OPL number of animals = 10; NOR number of animals = 10). (e–g) Memory measured by discrimination index of object exploration, and total exploratory behavior during testing in CaMK2A‐hM3D(Gq) + CNO (NOL number of animals = 12; OPL number of animals = 12; NOR number of animals = 12) vs. CaMK2A‐hM3D(Gq) + saline (NOL number of animals = 10; OPL number of animals = 10; NOR number of animals = 10). ns not significant, calculated by an unpaired *t*‐test. ^##^ *p* < 0.01, ^###^ *p* < 0.001, calculated by a one sample *t*‐test. Data are presented with box and whisker plots where “+” indicates the mean. Dashed line indicates no discrimination of objects (i.e., memory impairment).

We reported that genetic deletion of either Raptor (mTORC1 inhibition) or 4E‐BP2 (mTORC1 activation) in excitatory neurons does not impact memory formation (Huang et al. 2024). Consistently, here we confirmed that the modulation of mTORC1 activity in excitatory neurons via the regulation of neuronal activity following training (Figure 4a) did not impact long‐term memory in the NOL [CaMK2A‐hM4D(Gi): *p* = 0.2221, number of animals in saline group = 10, number of animals in CNO group = 9, *t* = 1.267, df = 17; CaMK2A‐hM3D(Gq): *p* = 0.7518, number of animals in saline group = 10, number of animals in CNO group = 12, *t* = 0.3206, df = 20, unpaired student's *t*‐test](Figure 4b,e), OPL [CaMK2A‐hM4D(Gi): *p* = 0.6145, *t* = 0.5130, df = 16.98; CaMK2A‐hM3D(Gq): *p* = 0.2510, *t* = 1.182, df = 19.96, unpaired student's *t*‐test](Figure 4c,f), and NOR [CaMK2A‐hM4D(Gi): *p* = 0.2836, *t* = 1.107, df = 17; CaMK2A‐hM3D(Gq): *p* = 0.6933, *t* = 0.4002, df = 20, unpaired student's *t*‐test](Figure 4d,g) tasks. The DI consistently remained significantly above baseline levels in both the CNO treatment group and the control group, and no changes in total exploratory behavior were observed (Figure 4b–g, right panels), showing that memory was intact. Taken together, the results demonstrate that manipulating neuronal activity and mTORC1 signaling in hippocampal excitatory neurons via hM4D(Gi) or hM3D(Gq)‐coupled DREADDs does not affect long‐term object memory and spatial memory.

The findings described here, together with our published results in conditional transgenic mouse models (Huang et al. 2024), show that mTORC1–4E‐BP1/2 signaling in inhibitory interneurons is crucial for long‐term memory, but is dispensable in excitatory neurons for hippocampus‐dependent memory.

## 4 Discussion

Protein synthesis controlled by mTORC1 is critical for synaptic plasticity and long‐term memory formation (Banko et al. 2005; Jobim et al. 2012; Pereyra et al. 2018; Tang et al. 2002; Zhu et al. 2018). The temporal requirement of mTORC1 for the consolidation of memory is well established by rapamycin treatment, an mTORC1 inhibitor, in the hippocampus (Myskiw et al. 2008; Pereyra et al. 2018). This intervention disrupts hippocampal‐dependent memory formation in mice, as determined by NOL and NOR tasks (Bekinschtein et al. 2007; Jobim et al. 2012; Lana et al. 2017; Myskiw et al. 2008; Pereyra et al. 2018; Qi et al. 2010; Tang et al. 2002; Zhu et al. 2018). Studies with cKO models underscore the crucial role of the mTORC1–4E‐BP2 axis in long‐term memory processes, particularly in inhibitory but not in excitatory neurons (Artinian et al. 2019; Huang et al. 2024; Khlaifia et al. 2022; Zhu et al. 2018). However, the impact of spatiotemporal modulation of mTORC1 signaling in inhibitory neurons on memory formation was not investigated. DREADDs provide a means to manipulate mTORC1 signaling via modulating neuronal activity with spatial and temporal precision (Roth 2016; Wess et al. 2013). In this study, we employed double‐floxed hM4D(Gi) and hM3D(Gq) DREADDs to either inhibit or activate Cre‐expressing inhibitory or excitatory neurons, specifically in the hippocampus of mice.

DREADDs are exogenous GPCRs that regulate neuronal activity and provide a model for understanding endogenous GPCR pathways. M3 Gα_q_‐GPCRs in hippocampal synapses activate phospholipase Cβ (PLCβ), leading to inositol triphosphate (IP3) and diacylglycerol (DAG) production, which increases intracellular calcium levels and activates protein kinase C (PKC) (Chen et al. 2017; de Munnik et al. 2015; Dickson et al. 2013). PKC has been shown to activate mTORC1 and increase p‐S6 levels in the brain (Blázquez et al. 2015; Cabezudo et al. 2021; Cheng et al. 2003; Pati et al. 2019; Xu et al. 2011). Similarly, artificial hM3D(Gq) activation promotes mTORC1 signaling both in cell culture and in vivo (Cabezudo et al. 2021; Gong et al. 2023; Shrestha, Ayata, et al. 2020; Shrestha, Shan, et al. 2020; Wu et al. 2020). The downstream signaling mechanisms of M4 Gα_i_‐GPCR pathway activation are context‐dependent and not fully understood. While M4 Gα_i_‐GPCR generally inhibits cAMP production and reduces PKA activity, it may also enhance cAMP production in specific cell types and under certain conditions, potentially inhibiting mTORC1 (Chen et al. 2017; Cooper 2003; de Munnik et al. 2015; El‐Armouche et al. 2003; Gilman 1995; Sobolczyk and Boczek 2022; Tang and Gilman 1991; Walker et al. 2017). For example, in insect Sf9 cells, Gα_i_‐GPCRs activate AC type II, allowing Gα_i_‐mediated activation of AC via Gα_s_‐GPCRs (βγ subunits) (Tang et al. 2002). A study in pancreatic α cells demonstrated that hM4D(Gi) activation does not alter p‐S6 levels (Gong et al. 2023). However, PKA has been shown to directly phosphorylate 4E‐BP1/2, suggesting a decrease in p‐4E‐BP1/2 levels upon Gα_i_‐GPCR activation (Lawrence Jr. et al. 1997). The effects of artificial hM4D(Gi) on mTORC1 signaling in the brain remain largely unexplored. Since hM4D(Gi) signaling suppresses neuronal activity, we expected that activation of hM4D(Gi) might decrease mTORC1 signaling (Beretta et al. 1996; Pearson et al. 1995). Our study is the first to address this knowledge gap regarding the neuron‐type‐specific impact of artificial hM3D(Gq) and hM4D(Gi) on mTORC1–4E‐BP1/2 signaling in vivo.

We report that hM4D(Gi) and hM3D(Gq) modulate mTORC1 downstream targets p‐S6 and p‐4E‐BP1/2 (mainly 4E‐BP2) in a cell‐type‐dependent manner. In inhibitory neurons, hM3D(Gq) activation increased p‐S6 and p‐4E‐BP1/2, while hM4D(Gi) activation reduced p‐S6 and p‐4E‐BP1/2. Importantly, activating either hM4D(Gi) or hM3D(Gq) in inhibitory neurons impaired memory. In sharp contrast, in excitatory neurons, hM4D(Gi) and hM3D(Gq) activation elicited only small changes in S6 phosphorylation (16%–22%), with no effect on p‐4E‐BP1/2 levels and memory formation. Further research is needed to determine the precise mechanisms linking DREADD‐associated neuronal activity to mTORC1 and to explore its effects on other signaling pathways in the brain—such as MAPK/ERK.

It is noteworthy that DREADDs activate other downstream signaling pathways in addition to mTORC1 (Huang et al. 2024; Ilyaskina et al. 2018). For example, hM4D(Gi) impacts cAMP signaling, and hM3D(Gq) impacts calcium dynamics and expression of the active form of ERK1/2 (Alcacer et al. 2017; Armbruster et al. 2007; de Vargas et al. 2017; Ganguly and Lee 2013; Lee 2015; Wilmott and Thompson 2013). Our study cannot rule out possible effects via these mechanisms. One possible approach to demonstrate that DREADDs disrupt memory formation specifically via mTORC1–4E‐BP2 in inhibitory neurons would entail showing that DREADDs have no effect on memory in Raptor or 4E‐BP2 cKO mouse models. However, memory is impaired by the cKO of Raptor or 4E‐BP2 in inhibitory neurons (Huang et al. 2024). Techniques like cell‐type‐specific and drug‐inducible protein synthesis inhibition (ciPSI) or optogenetic tools like Opto4E‐BP could provide direct validation of the role of cell‐type‐specific protein synthesis in memory (Alapin et al. 2023; Shrestha, Ayata, et al. 2020; Shrestha, Shan, et al. 2020). By decreasing eIF4E‐dependent translation in situ in specific neuron subtypes, these methods provide an understanding of spatiotemporal memory formation. In addition, how signaling pathways are modulated by hM4D(Gi) and hM3D(Gq) activation, such as ERK1/2, PI3K, and AMP‐activated protein kinase (AMPK), should be investigated by cell‐type‐specific genetic editing methods or advanced optogenetic tools (Alcacer et al. 2017; Guo et al. 2019; Meister et al. 2021; Okamoto et al. 2018; Thompson et al. 2018).

Unlike the transgenic models, chemogenetic modulation of inhibitory neuronal mTORC1–4E‐BP1/2 signaling did not completely ablate memory, particularly in the NOR test. This partial effect is likely due to the involvement of other brain regions, such as the dentate gyrus and prefrontal cortex, in memory formation (Barker and Warburton 2011; Dees and Kesner 2013). Supporting this result, previous research has shown that hM4D(Gi) activation in dorsal hippocampal excitatory neurons does not impair long‐term NOR memory at 24 h (Tuscher et al. 2018). While our study highlights the crucial role of inhibitory neuronal activity and mTORC1 signaling in object location and recognition memory, we did not observe a significant role for excitatory neurons, which contradicts some prior research. For example, hM4D(Gi) activation in the ventral hippocampal excitatory neurons was shown to disrupt contextual fear memory consolidation and extinction (Gong et al. 2022; Terranova et al. 2022; Zhu et al. 2014). hM3D(Gq) activation in excitatory neurons of the mouse amygdala and piriform cortex impairs contextual fear memory and spatial memory, respectively (Levitan et al. 2020; Shrestha and Klann 2022; Shrestha, Shan, et al. 2020; Wang et al. 2022). Recent work has further shown that fear conditioning requires hippocampal CaMK2A^+^excitatory neurons and SST^+^ interneurons to undergo profound reconfiguration of proteostatic control, while interneurons primarily modify their synaptic transmission (Oliveira et al. 2025). This difference may reflect distinct neural circuit involvement—with contextual fear memory relying more on excitatory neuron activity—whereas object memory requires GABAergic interneuron modulation. Oliveira et al. (2025) found that PValb^+^ and SST^+^ interneurons exhibited a significant association between learning and translatome changes during the early phase of memory consolidation. Meanwhile, excitatory neurons have an immediate early dynamic translational response that decreases later. It can be argued that CNO injected by i.p. will require a certain amount of time to reach the hippocampus when changes in mTORC1 activity in excitatory neurons may no longer impact memory. Instead, interneurons may play a key role in memory‐related translational changes, a process likely regulated by mTORC1. Taken together with our data, these findings collectively suggest that excitatory and inhibitory neurons engage different molecular pathways for different types of memory, particularly regarding mechanisms of translational control.

In the memory tests, behaviorally trained mice likely already have alterations in mTORC1 activity in response to the new stimulus, as this pathway is required for memory formation. Since we demonstrated that mTORC1 activity can be bi‐directionally modulated using DREADDs in untrained mice, we anticipate that activating DREADDs immediately after the learning stimulus (i.e., training) allowed us to modulate neuronal activity and interfere with changes in endogenous mTORC1 signaling during the normal learning and memory process. Since either inhibiting or enhancing mTORC1 in inhibitory neurons prevented memory formation, we argue that inhibitory neurons require an optimal level of mTORC1 signaling for memory formation. These results are consistent with the findings of our previous study and together provide strong evidence for this model (Huang et al. 2024). Future studies are required to directly assess mTORC1 activity in inhibitory neurons after training compared to naive mice to fully understand the precise training‐induced changes in mTORC1 activity.

Another possible explanation for the different effects observed in the memory tests following excitatory neuronal modulation is the timing and different dosages of CNO administration. It is notable that DREADDs activation by CNO, confounding effects on neuronal activity and animal behaviors are dose‐ and dosing frequency‐dependent (Pati et al. 2019; Zhu et al. 2014). In addition, the efficacy of DREADDs activation may vary depending on the timing of CNO administration relative to the training or testing phase, as well as the dosage administered. To ensure precise modulation of neuronal activity while minimizing off‐target effects on electrophysiology and general behavior, we used 1 post‐training low CNO dose of 0.1 mg/kg (Alexander et al. 2009; Rogan and Roth 2011; Vaidyanathan et al. 2021). The low dosage we used strengthens the reliability of our findings by isolating the specific effects of neuronal modulation on memory outcomes (Gong et al. 2022; Terranova et al. 2022; Zhu et al. 2014). Taken together, expanding the application of DREADDs across brain regions and administering CNO at different dosages and at different time points during learning and memory processes could provide deep insights into the cellular signaling mechanisms underlying memory.

It is still unclear what mechanisms explain the differences between excitatory and inhibitory neurons. It is conceivable that mTORC1 affects different gene expression profiles in excitatory and inhibitory neurons. Indeed, single‐cell RNA‐seq (scRNA‐Seq) analysis of hypothalamic pro‐opiomelanocortin neurons has revealed that GABAergic and glutamatergic subpopulations are molecularly distinct, although containing the same mTORC1 pathway components (Saucisse et al. 2021). This observation corroborates our published findings, indicating the essential role of mTORC1 and 4E‐BP2 in inhibitory neurons for the establishment of long‐term memory (Huang et al. 2024). To further investigate the reasons for the different functional outcomes of manipulating the mTORC1 signaling axis through modulation of neuronal activity in excitatory versus inhibitory neurons, scRNA‐Seq studies will be needed (Saucisse et al. 2021).

Understanding how dysregulated mTORC1–4E‐BP1/2 signaling and translation affects distinct brain cell types is essential for uncovering the mechanisms underlying neurological disorders and for developing therapeutic strategies. Dysregulation of the mTORC1–4E‐BP1/2 axis has been linked to diseases such as autism, epilepsy, and Alzheimer's disease (AD) (Aguilar‐Valles et al. 2021; Bermudez et al. 2024; Ribeiro et al. 2024; Sharma et al. 2021; Wiebe et al. 2019). Our previous findings, together with the results of the present study, demonstrate that optimal mTORC1–4E‐BP1/2 activity in inhibitory neurons is required for memory consolidation (Huang et al. 2024). Recent studies indicate that targeting 4E‐BP1/2 to restore translation regulation may offer a promising strategy for restoring memory function in AD (Bermudez et al. 2024; Ribeiro et al. 2024). Specifically, 4E‐BP1 inactivation in microglia enhances mitochondrial metabolism and promotes neuroprotection, while 4E‐BP2 deletion prevents amyloid‐β oligomer‐induced suppression of protein synthesis and rescues memory deficits in AD mouse models.

In conclusion, activating either hM4D(Gi)‐ or hM3D(Gq)‐ DREADDs in hippocampal inhibitory, but not excitatory neurons, engenders long‐term memory deficits. These findings underscore the critical role of inhibitory neuronal activity and mTORC1–4E‐BP1/2 signaling in memory formation, which highlights the importance of cell‐type‐specific mRNA translational control of memory.

## Author Contributions

**Ziying Huang:** conceptualization, investigation, writing – original draft, writing – review and editing, methodology, data curation, validation. **Niaz Mahmood:** writing – review and editing. **Jean‐Claude Lacaille:** writing – review and editing. **Shane Wiebe:** writing – review and editing, conceptualization, methodology, writing – original draft, supervision. **Nahum Sonenberg:** supervision, writing – review and editing, funding acquisition.

## Ethics Statement

All animal procedures and experiments were performed in accordance with the McGill University Facility Animal Care Committee (FACC) and the Canadian Council on Animal Care (CCAC) regulations.

## Consent

All authors have given their consent for publication.

## Conflicts of Interest

The authors declare no conflicts of interest.

## Supporting information


Figure S1.

## Data Availability

The datasets and analyses supporting the findings of this study are available from the corresponding author upon reasonable request.
