# Supplementary figures and images for: Hippocampal Inhibitory Interneuron‐Specific DREADDs Treatment Alters mTORC1‐4E‐BP Signaling and Impairs Memory Formation
Source: J Neurochem. 2025 Mar 24;169(3):e70048. doi: 10.1111/jnc.70048 (PMC11931476; doi:10.1111/jnc.70048)

Supplemental Fig. 1

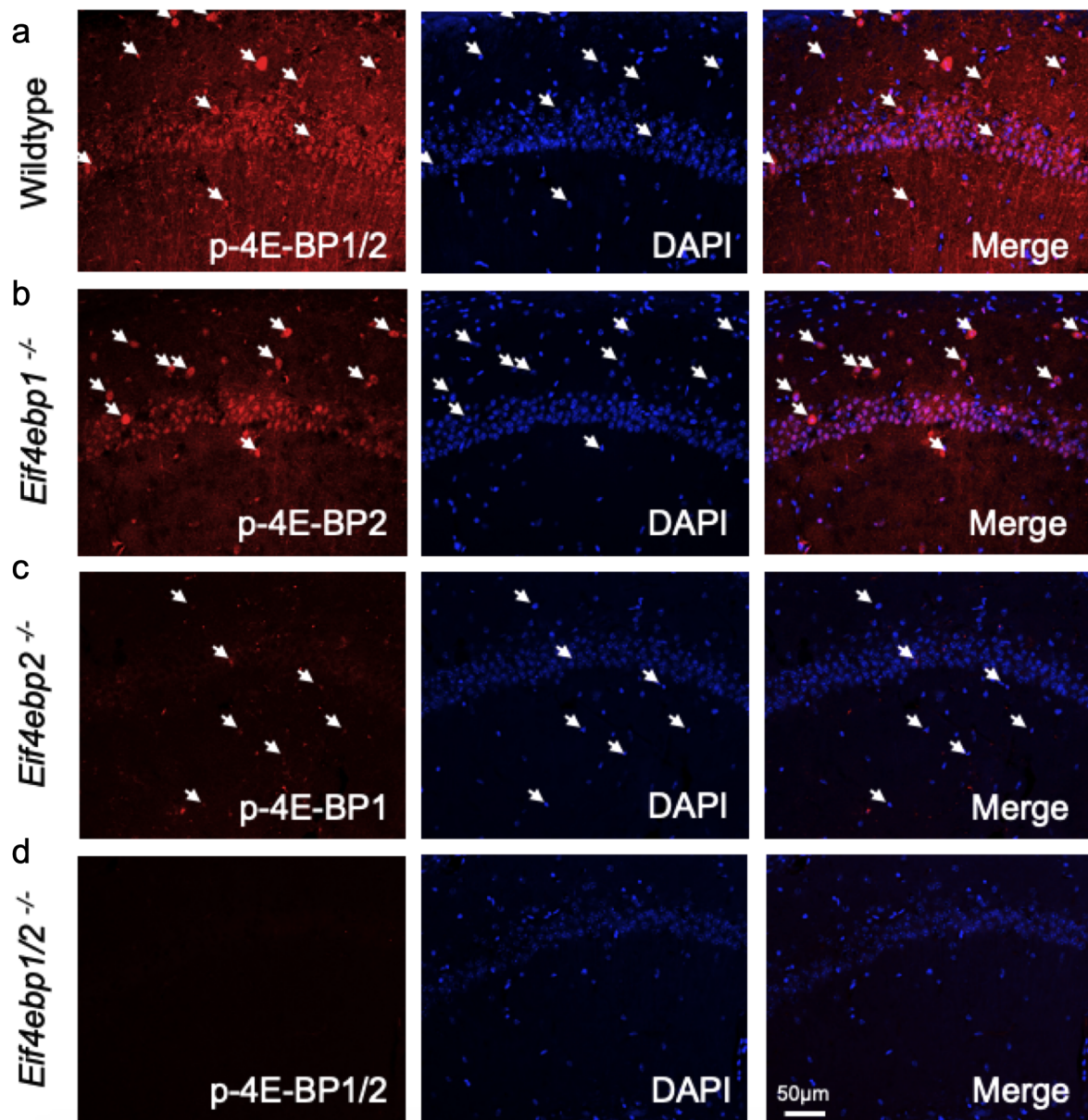

Supplement: Supplementary file 1 — Figure S1. [file JNC-169-0-s001.pdf]
